# Heterogeneity in peripheral blood immune lymphocyte subsets predicts the response of immunotherapy or chemoradiotherapy in advanced lung cancer: an analysis across different pathological types, treatment modalities and age

**DOI:** 10.3389/fimmu.2024.1464728

**Published:** 2024-10-17

**Authors:** Chuanwang Miao, Yuanji Chen, Hao Zhang, Wei Zhao, Cunliang Wang, Zeliang Ma, Shan Zhu, Xudong Hu

**Affiliations:** ^1^ Department of Radiation Oncology, Shandong Cancer Hospital and Institute, Shandong First Medical University and Shandong Academy of Medical Sciences, Jinan, Shandong, China; ^2^ Department of Clinical Medicine, Shandong First Medical University, Jinan, Shandong, China; ^3^ Department of Radiotherapy, Linyi Cancer Hospital, Linyi, Shandong, China; ^4^ Department of Radiation Oncology, Cancer Hospital, Chinese Academy of Medical Sciences and Peking Union Medical College, Beijing, China; ^5^ Department of Oncology, Mayo Clinic, Rochester, MN, United States; ^6^ Department of Radiation Oncology, Shandong Provincial ENT Hospital, Shandong University, Jinan, Shandong, China

**Keywords:** radiotherapy, peripheral blood circulation, prognosis, lung cancer, lymphocyte subsets

## Abstract

**Background:**

The shaping of the tumor immune microenvironment does not only rely on tumor-infiltrating lymphocytes but on the recruitment of lymphocytes in peripheral blood. Monitoring peripheral blood lymphocyte subsets level (PBLSL) can predict treatment response and prognosis with immune checkpoint inhibitors. This study investigated the heterogeneity of PBLSL in response to chemoradiotherapy (CRT) or combined with immunotherapy (CRIT) in advanced lung cancer patients.

**Methods:**

77 patients with advanced lung cancer receiving CRT or CRIT were divided into treatment-responsive and non-responsive groups based on efficacy. The study analyzed short-term efficacy and progression-free survival (PFS) according to baseline PBLSL and explored the impact under different stratifications, including treatment modality, pathology type, and age.

**Results:**

In all patients, higher levels of B cells, higher CD4+/CD8+ T cell ratios, and lower CD8+ T cell levels were associated with better short-term outcomes (P = 0.0035, P = 0.044, P = 0.022). Subgroup analysis revealed that in the CRT group, higher B cell levels correlated with improved efficacy (P = 0.011) and superior PFS (P = 0.048, HR = 0.3886, 95% CI = 0.1696 to 0.8902). In the CRIT group, higher CD4+ T cell levels, lower CD8+ T cell levels, and higher CD4+/CD8+ T cell ratios were linked to better efficacy (P = 0.038, P = 0.047, P = 0.017). For adenocarcinoma patients, higher CD4+/CD8+ T cell ratios and lower CD8+ T cell levels predicted better efficacy (P = 0.0155, P = 0.0119). B cell levels were significant in squamous cell carcinoma (P = 0.0291), while no PBLSL was predictive for small cell lung cancer. Among patients under 65, higher B cell levels were linked to improved efficacy and prolonged PFS (P = 0.0036, P = 0.0332, HR = 0.4111, 95% CI = 0.1973 to 0.8563). For patients over 65, differences in CD4+ T cell levels and CD4+/CD8+ T cell ratios were significant (P = 0.0433, P = 0.0338).

**Conclusion:**

PBLSL predicted efficacy and prognosis in various patient stratifications, suggesting PBLSL is a reliable predictor for CRT and CRIT in advanced lung cancer. Detecting different cellular subpopulations helps identify patients with significant treatment responses across different stratifications.

## 1 Introduction

Radiotherapy remains a cornerstone in the treatment of advanced lung cancer, regardless of the specific pathology. For many years, the standard of care has been chemoradiotherapy (CRT), combining chemotherapy with radiotherapy (1–4). Immune checkpoint inhibitors (ICIs) have ushered in a new era in cancer treatment. ICIs represented by programmed cell death protein 1 (PD-1) inhibitors and programmed cell death protein ligand 1 (PD-L1) inhibitors have demonstrated enduring, favorable objective responses in patients diagnosed with both non-small cell lung cancer (NSCLC) and small cell lung cancer (SCLC) (5, 6). ICIs have been benchmark drugs in the standard treatment of advanced lung cancer.

Besides its local tumor-killing effects, radiotherapy can stimulate immune activation, resulting in systemic changes in immune cell populations within the tumor microenvironment (7, 8), forming the foundational theory behind combining immunotherapy and radiotherapy. The PACIFIC study, a phase III randomized controlled trial of the PD-L1 inhibitor Durvalumab consolidation therapy versus placebo, showed that the Durvalumab consolidation therapy group had significantly better progression-free survival (PFS) than the placebo group after concurrent chemoradiotherapy (9, 10). In the GEMSTONE-301 study, Sugemalimab consolidation therapy significantly prolonged PFS versus placebo in patients who had not progressed on sequential chemoradiation or concurrent radiotherapy (11). Sugemalimab has been approved by the National Medical Products Administration for use in inoperable locally advanced NSCLC as a consolidation therapy after concurrent or sequential chemoradiation therapy.

While standard treatment involving immune consolidation therapy and chemoradiotherapy extends survival in patients with advanced lung cancer, approximately 40% of patients continue to experience disease progression. Reliable biomarkers for predicting efficacy and prognosis in CRT and CRIT are still lacking.

Tumor recurrence and metastasis are intricately associated with compromised immune function. Impaired immunity enables tumors to evade immune responses, thereby worsening tumor progression and dissemination, establishing a vicious cycle. Lymphocytes are critical in regulating the immune system and targeting tumor cells for destruction. The intricate interplay among lymphocyte subsets constitutes the foundation of the immune system and profoundly impacts the body’s disease response (12). Radiotherapy stimulates the generation of *in situ* tumor vaccines and mobilizes tumor-infiltrating lymphocytes (TILs), thereby enhancing the tumor microenvironment (13, 14).

After ionizing radiation, dendritic cells capture destroyed tumor cells by releasing tumor-associated antigens, thus serving as an *in-situ* tumor vaccine. This activation induces systemic immunogenicity and distant effects (15, 16). However, solely depending on the activation of tumor-resident TILs fails to fully elucidate the unified response mechanism that underlies distant effects across various metastatic tumors, which means peripheral immune cells likely play a vital role in clinical responses (17). A study discovered that patients benefiting from ICIs treatment experienced significant proliferation of PD-1-positive CD8+ T cells in peripheral blood, whereas patients with disease progression exhibited delayed or absent responses in these cells (18). Single-cell RNA sequencing and T-cell receptor repertoire sequencing experiments unveiled that the clonal replacement of exhausted CD8+ T cells in tumors does not stem from the reactivation of pre-existing TILs but chiefly arises from newly recruited cells in the peripheral blood (19). Thus, extensive T cell proliferation may originate not only from pre-existing TILs within tumors but also from recruited T cells in peripheral blood.

Although lymphocytes near tumor tissue could provide a more accurate reflection of tumor biology, tissue biopsy is hindered by the heterogeneity across various tumor regions. Moreover, the invasive nature of biopsies and the impracticality of repetitive serial examinations make them unsuitable for advanced lung cancer patients (20). Obtaining lymphocyte subsets from peripheral blood circumvents these challenges and allows repeated dynamic monitoring. Peripheral blood lymphocytes can reflect the body’s overall immune status. Several studies have shown that changes in the peripheral blood lymphocyte subset correlate with the efficacy of tumor immunotherapy (21–24). However, no studies have yet explored the influence of pre-treatment levels of peripheral blood lymphocyte subsets on standard chemoradiotherapy and subsequent immune consolidation therapy. This study collected pre-treatment distributions of peripheral blood lymphocyte subsets level (PBLSL) in unresectable lung cancer patients who received CRT or CRIT to assess their predictive potential for treatment efficacy and prognosis.

## 2 Methods

### 2.1 Baseline of the patient

We included 77 patients diagnosed with advanced lung cancer and admitted from January 2, 2019, to December 26, 2022, from Shandong Cancer Hospital, who underwent CRT or CRIT. We collected pre-treatment clinical data from patients, including gender, age, pathology type, tumor stage, and treatment regimen. Pre-treatment lymphocyte subset data were obtained through flow cytometry. These data served as baseline characteristics for all patients. Informed consent was obtained from all patients in accordance with the Declaration of Helsinki.

### 2.2 Inclusion criteria

Inclusion criteria were as follows: (1) Diagnosis of lung cancer was confirmed by histopathology or cytology. (2) Patients were clinically graded as advanced according to the American Joint Committee on Cancer (AJCC) 8th edition staging system and the American Legion (VALG) stage II staging system. (3) The age is 18 or older and has an Eastern Cooperative Oncology Group (ECOG) physical status score of 0-1. (4) Patients should not have received standard antitumor therapy.

### 2.3 Exclusion criteria

Patients should be excluded if the following conditions exist: (1) Failure to submit pre-treatment peripheral blood lymphocyte subset results. (2) Failure to complete a treatment cycle or interruption of treatment leading to exclusion. (3) Patients who are pregnant or lactating. (4) Presence of psychiatric disorders that prevent cooperation with treatment (5) Patients with a combination of severe pneumonia or other respiratory infections or hemato-oncologic and autoimmune diseases.

### 2.4 Flow cytometry

Blood samples were collected via peripheral venipuncture from each participant within the initial one week before therapy commenced. Blood was treated with erythrocyte lysis buffer to remove red blood cells, leaving a suspension of leukocytes. Staining protocol: the leukocytes were then stained with fluorochrome-conjugated antibodies. The antibodies were sourced from Tongshengshidai (Z6410010, Z6410002; Beijing, China), Beckman (6607073; Brea, CA, USA), and BD (665343; Franklin Lakes, NJ, USA). Staining was conducted according to the manufacturer’s protocols. Flow cytometry analysis: following staining, cells were analyzed using a flow cytometer (Beckman Coulter DxFLEX, Brea, CA, USA). These samples were analyzed to determine the proportions of various PBLSL, including CD3−CD56+ [natural killer (NK) cells], CD3−CD19+ (B cells), CD3+ (total T lymphocytes), CD3+CD4+ [helper T (Th) cells], and CD3+CD8+ [cytotoxic and suppressor T (Tc/Ts) cells], along with the CD4+/CD8+ ratio.

### 2.5 Treatment and follow−up

All CRT patients with NSCLC and SCLC received standard treatment, including platinum-containing two-agent chemotherapy and intensity-modulated radiation therapy, which were recommended by treatment guidelines. Patients who received CRIT extra completed at least two cycles of ICIs.

Evaluation of initial disease response after therapy is a priority. Imaging data of CRT cases were collected four weeks after the end of treatment. Patients who received CRIT were selected for imaging data after completing two cycles of ICIs. Data included CT and PET-CT scans that allowed the interpretation of solid tumors. At Least two senior radiologists separately evaluated imaging results according to Response Evaluation Criteria in Solid Tumors (RECIST) version 1.1. The complete disappearance of the target lesions was evaluated as a complete response (CR), a reduction of > 30% in total target lesions was evaluated as a partial response (PR), a reduction of < 30% or an increase of < 20% in the sum of target lesions was assessed as stable disease (SD). An increase of > 20% in total target lesions was evaluated as progressive disease (PD). CR and PR were considered responsive, whereas SD and PD denoted non-responsiveness. PFS was defined as the duration from initial treatment to disease progression or death from any cause.

### 2.6 Statistical analysis

The normality of statistical data was assessed by the Shapiro-Wilk test. Data following a normal distribution were expressed as mean ± standard deviation (mean ± SD), while skewed data were expressed as median or range. Normally distributed data were assessed with a t-test and non-normal data with a rank sum test. One-way ANOVA analyzed differences among three or more groups of normally distributed variables with *post-hoc* Bonferroni and Tamhane tests. Enumeration data were presented as absolute numbers and compared using the chi-square test (χ² test) or Fisher’s exact test. Receiver operating characteristic (ROC) curves assessed the predictive ability of peripheral blood lymphocyte subsets for efficacy, with the optimal cut-off point determined by the Youden Index. ROC is then used to evaluate its performance using 5-fold cross-validation. Kaplan-Meier curves plotted PFS and were compared using the log-rank test. Hazard ratios (HR) were presented with 95% confidence intervals (CI). Two-tailed tests defined statistical significance as P < 0.05. Data analysis used IBM SPSS 28.0 (IBM Corporation, USA) and R 4.4.0 (The R Foundation), and visualizations were generated with GraphPad Prism 9.0 (San Diego, USA), ensuring statistical analysis supported the findings and facilitated interpretation.

## 3 Results

**Table 1** has summarized the baseline characteristics of the 77 patients, including gender, age, histological type, and radiotherapy received, presented as mean ± standard deviation or median (range). **Table 2** showed the relationship between PBLSLs and pre-treatment baseline characteristics. No significant differences in PBLSLs have indicated well-matched samples, minimizing group differences’ impact on analysis. Ultimately, 48 patients have been in the responder group, and 29 have been in the non-responder group.

**Table 1:** Baseline characteristics of patients.

Characteristics	N (Range, Median)	%
Gender		
Male	55	71.4
Female	22	28.6
Age		
≤ 65	57 (45-65, 57)	74.0
> 65	20 (66-85, 70)	26.0
Histology		
Adenocarcinoma	31	40.0
Squamous cell carcinoma	23	30.0
Small-cell carcinoma	23	30.0
Treatment		
CRT	46	59.7
CRIT	31	40.3
ICI		
PD-1 inhibitor	10	32.3
PD-L1 inhibitor	21	67.7
Lymphocyte subsets (%)	Mean	± SD or Range
CD3-CD16+CD56+ (NK)	20.85	4.9-57.0
CD3-CD19+ (B)	10.72	± 5.70
CD3 (Total T)	66.60	31.2-94.7
CD3+CD4+ (Th)	39.33	± 9.84
CD3+CD8+ (Tc/Th)	22.66	11.3-61.0
CD4+/CD8+	2.01	± 0.923

CRT, chemoradiotherapy; CRIT, chemoradiotherapy and immunotherapy.

**Table 2:** Correlation between patients’ baseline characteristics and lymphocyte subsets.

	CD3CD16+CD56+ (NK)	P	CD3-CD19+ (B)	P	CD3 (Total T)	P	CD3+CD4+ (Th, CD4+)	P	CD3+CD8+ (Tc/Ts, CD8+)	P	CD4+/CD8+	P
Gender												
Male	18.20(5.0-57.0)	0.791	10.12 ± 5.45	0.109	68.60(31.2-94.7)	0.852	38.48 ± 10.57	0.116	21.00(11.3-61.0)	0.114	1.91 ± 0.94	0.057
Female	19.55(4.9-41.1)		12.23 ± 6.18		65.90(47.7-89.2)		41.46 ± 7.53		18.60(11.3-34.3)		2.28 ± 0.84	
Age												
≤65	18.80 (4.9-52.1)	0.723	10.59 ± 5.85	0.767	66.20 (31.5-94.7)	0.875	39.54 ± 9.25	0.760	20.50 (11.3-61.0)	0.684	2.04 ± 0.90	0.715
> 65	17.15 (8.9-57.0)		11.09 ± 5.40		67.35 (31.2-86.9)		38.75 ± 11.59		21.25 (12.3-43.8)		1.95 ± 1.01	
Treatment												
CRIT	18.80(4.9-57.0)	0.979	10.30 ± 6.52	0.517	66.6(31.2-94.7)	0.861	37.22 ± 9.89	0.122	21.00(11.3-61.0)	0.131	1.8019 ± 0.97	0.099
CRT	18.45(7.4-48.8)		11.00 ± 5.14		65.9-(31.5-89.2)		40.76 ± 9.65		20.05(11.3-43.8)		2.16 ± 0.87	
Histology												
Squamous cell carcinoma	17.65 (8.5-57.0)	0.709	9.24 ± 3.68	0.591	67.55 (31.2-31.2)	0.577	41.33 ± 10.19	0.312	21.40 (12.3-35.5)	0.336	1.98 ± 0.83	0.536
Adenocarcinoma	19.00 (4.9-45.7)		10.67 ± 6.19		64.50 (31.5-89.2)		37.04 ± 10.34		23.10 (11.3-43.8)		1.86 ± 1.04	
Small-cell lung cancer	20.10 (5.0-52.1)		11.27 ± 5.95		65.68 (37.2-94.7)		39.52 ± 8.30		19.20 (11.3-61.0)		2.15 ± 0.79	

CRT, chemoradiotherapy; CRIT, chemoradiotherapy and immunotherapy.

### 3.1 The correlation between PBLSL and efficacy of all patients

**Figure 1:**
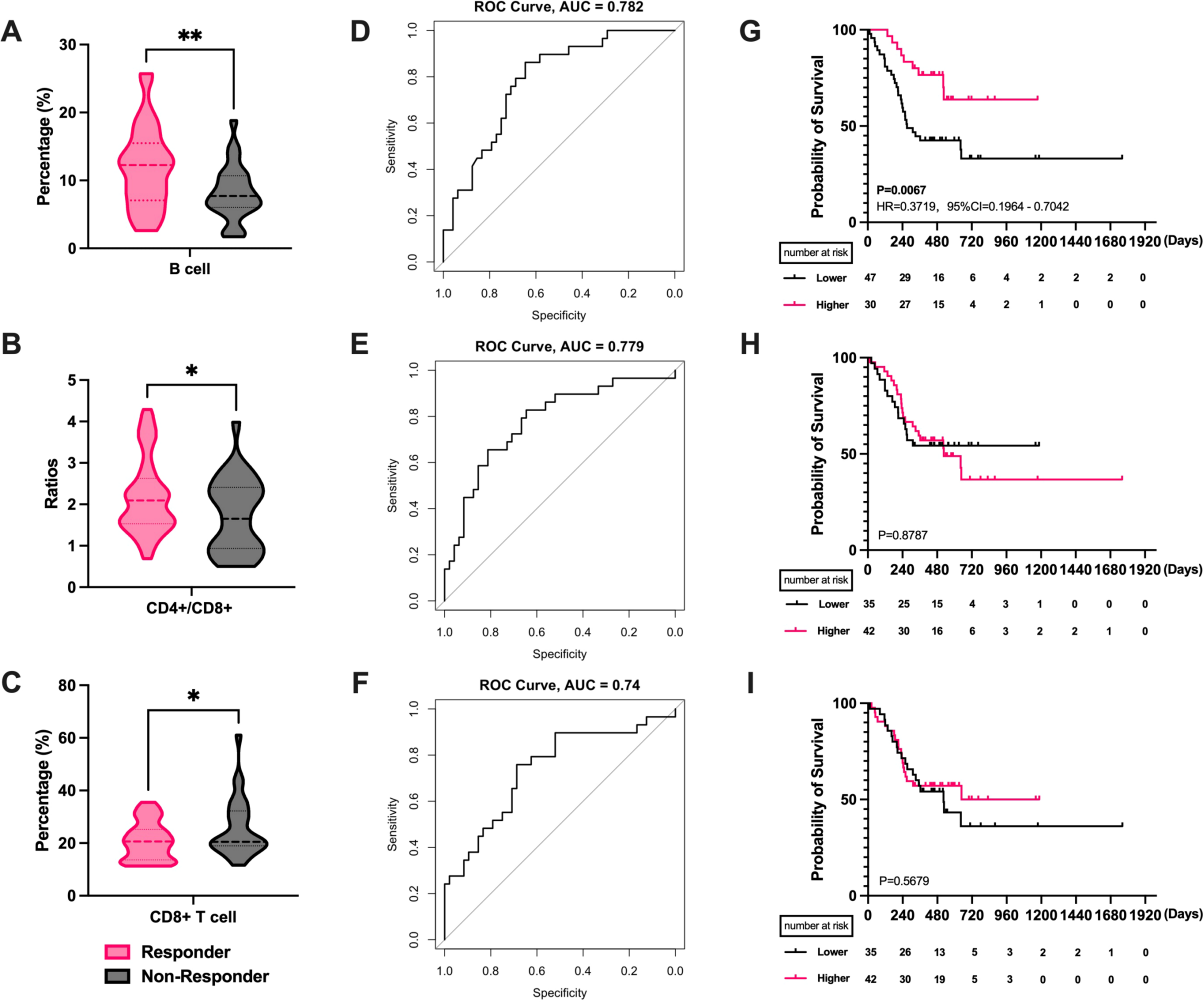
The correlation between PBLSL and efficacy of all patients. **(A)** B cell level between responders and non-responders. **(B)** CD4+/CD8+ T cell ratios between responders and non-responders. **(C)** CD8+ T cell level between responders and non-responders. **(D)** The ROC curves of B cell baseline levels. **(E)** The ROC curves of CD4+/CD8+ T cell ratios. **(F)** The ROC curves of CD8+ T cell levels. **(G)** PFS between the high and low B cell level groups. **(H)** PFS in the groups of high and low CD4+/CD8+ T cell ratios. **(I)** PFS between the high and low CD8+ T cell level groups. *p<0.05; **p<0.01; PBLSL, peripheral blood lymphocyte subsets level; PFS, progression-free survival.

We first analyzed the effects and prognosis of various PBLSLs on all patients. The results have shown a correlation between radiotherapy efficacy in 77 patients and pre-treatment levels of B cells (P = 0.0035, **Figure 1A**), CD4+/CD8+ T cell ratios (P = 0.044, **Figure 1B**), and CD8+ T cells (P = 0.022, **Figure 1C**). Responders exhibited higher proportions of B cells and CD4+/CD8+ T cell ratios, with lower CD8+ T cell levels. No significant differences were found among other lymphocyte subgroups. ROC curves assessed the relationship between these lymphocyte subsets and radiotherapy efficacy. ROCs showed that B cell levels predicted treatment efficacy (AUC = 0.782, **Figure 1D**), as did the CD4+/CD8+ T cell ratio (AUC = 0.779, **Figure 1E**) and CD8+ T cells (AUC = 0.74, **Figure 1F**). Additionally, all the mean AUC of the cross-validation were above 0.65, indicating stable predictive ability (**Supplementary Figure S1**).

Patients were stratified into high and low-level groups based on Youden index value. Kaplan-Meier curves showed a median PFS of 272 days for the low-level group, while the median PFS for the high-level group was not reached. The difference in B cell levels indicated favorable prognostic predictive ability (P = 0.0067, HR = 0.3719, 95% CI = 0.1964 to 0.7042, **Figure 1G**). Similarly, CD4+/CD8+ T cell ratio and CD8+ T cells were categorized into high and low level groups. However, no significant differences were observed in the survival curves for CD4+/CD8+ T cell ratios (P = 0.878, **Figure 1H**) and CD8+ T cells (P = 0.567, **Figure 1I**).

### 3.2 The correlation between PBLSL and efficacy of different treatment modality groups

**Figure 2:**
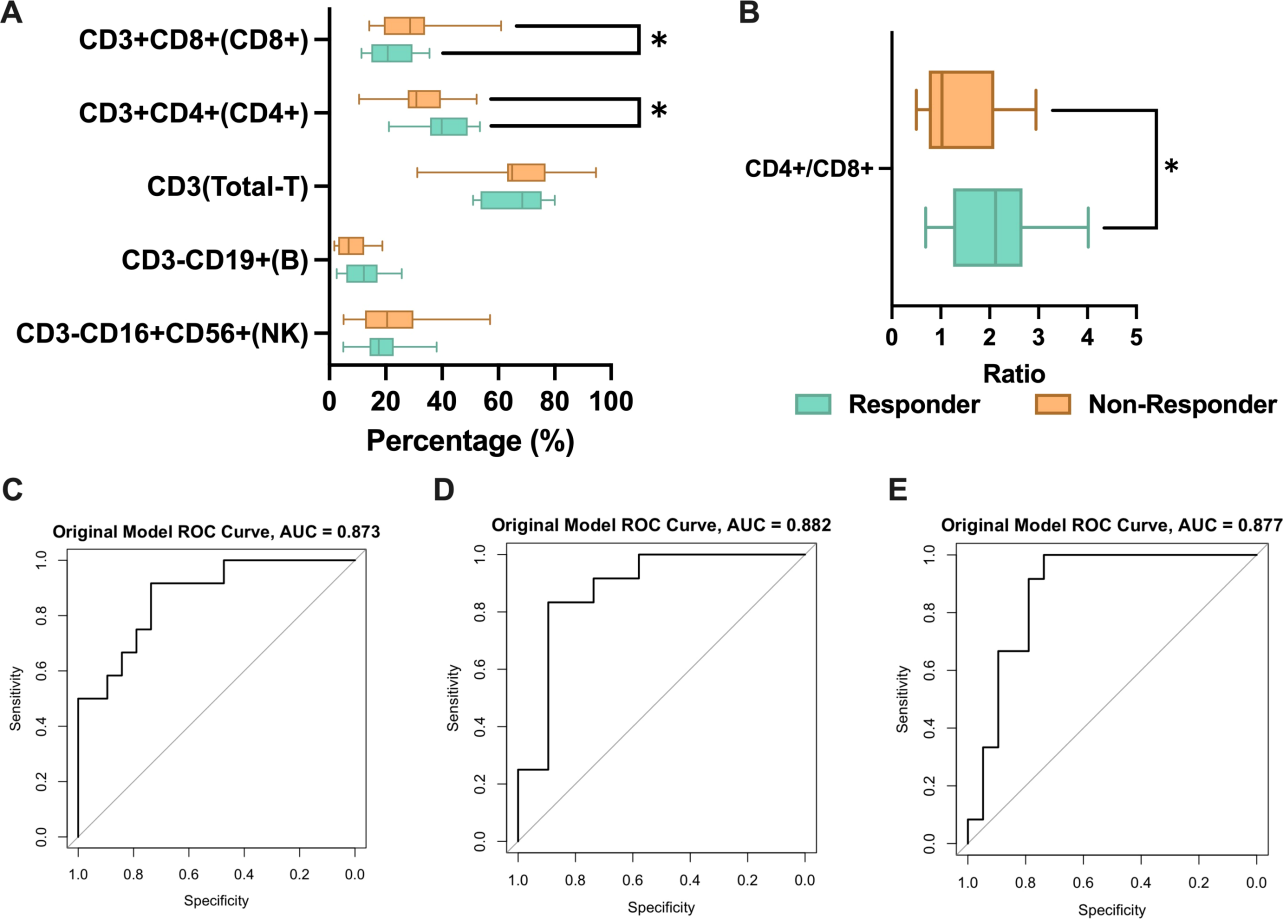
The correlation between PBLSL and efficacy of CRIT group. **(A)** PBLSL between responders and non-responders of patients undergoing CRIT. **(B)** CD4+/CD8+ T cell ratios between responders and non-responders. **(C)** The ROC curves of CD4+ T cell levels. **(D)** The ROC curves of CD4+/CD8+ T cell ratios. **(E)** The ROC curves of CD8+ T cell levels. *p<0.05; CRIT, chemoradiotherapy and immunotherapy.

**Figure 3:**
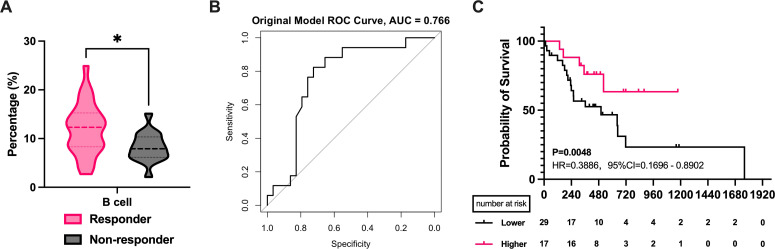
The correlation between PBLSL and efficacy of CRT group. **(A)** B cell level between responders and non-responders. **(B)** The ROC curves of B cell baseline levels. **(C)** PFS between the high and low B cell level groups. *p<0.05; CRT, chemoradiotherapy.

31 individuals received CRIT: 10 received PD-1 inhibitors, and 21 received PD-L1 inhibitors. The comparison showed that the treatment-benefited group had higher CD4+ T cell levels (P = 0.038, **Figure 2A**), lower CD8+ T cell levels (P = 0.047, **Figure 2A**), and a higher CD4+/CD8+ T cell ratios (P = 0.017, **Figure 2B**). ROC curve analysis revealed significance for CD4+ T cells (AUC=0.873, **Figure 2C**), CD4+/CD8+ T cell ratios (AUC=0.882, **Figure 2D**) and CD8+ T cells(AUC=0.877, **Figure 2E**). But cross-validation indicates that the mean AUC for CD8+ is less than 0.6. This suggests that CD8+ may not have stable predictive ability (**Supplementary Figure S2**). Optimal cut-off values for CD4+ T cell levels and CD4+/CD8+ T cell ratios were determined as 33.45 and 1.185, respectively, using the Youden index. For Kaplan-Meier curve analysis, patients were categorized into high and low-level/ratio groups. CD8+ T cells, lacking significance in ROC analysis, were categorized using median values for survival curve plotting. Unfortunately, no correlation was found between peripheral blood lymphocyte subset levels and progression-free survival (**Supplementary Figure S3**). We found that differences in B cell levels were associated with the efficacy of the CRT group (P = 0.011, **Figure 3A**). Higher B cell levels were correlated with better treatment efficacy. No significant differences were found among other lymphocyte subgroups. Further, B cell levels predicted therapeutic efficacy (AUC = 0.766, **Figure 3B**). Mean AUC for B cell Is 0.667 (**Supplementary Figure S2**). Kaplan-Meier curves showed a median PFS of 501 days for the low-level group, while the median PFS for the high-level group was not reached. The difference in B cell levels indicated favorable prognostic predictive ability (P = 0.048, HR = 0.3886, 95% CI = 0.1696 to 0.8902, **Figure 3C**).

### 3.3 The correlation between PBLSL and efficacy of different pathology type groups

**Figure 4:**
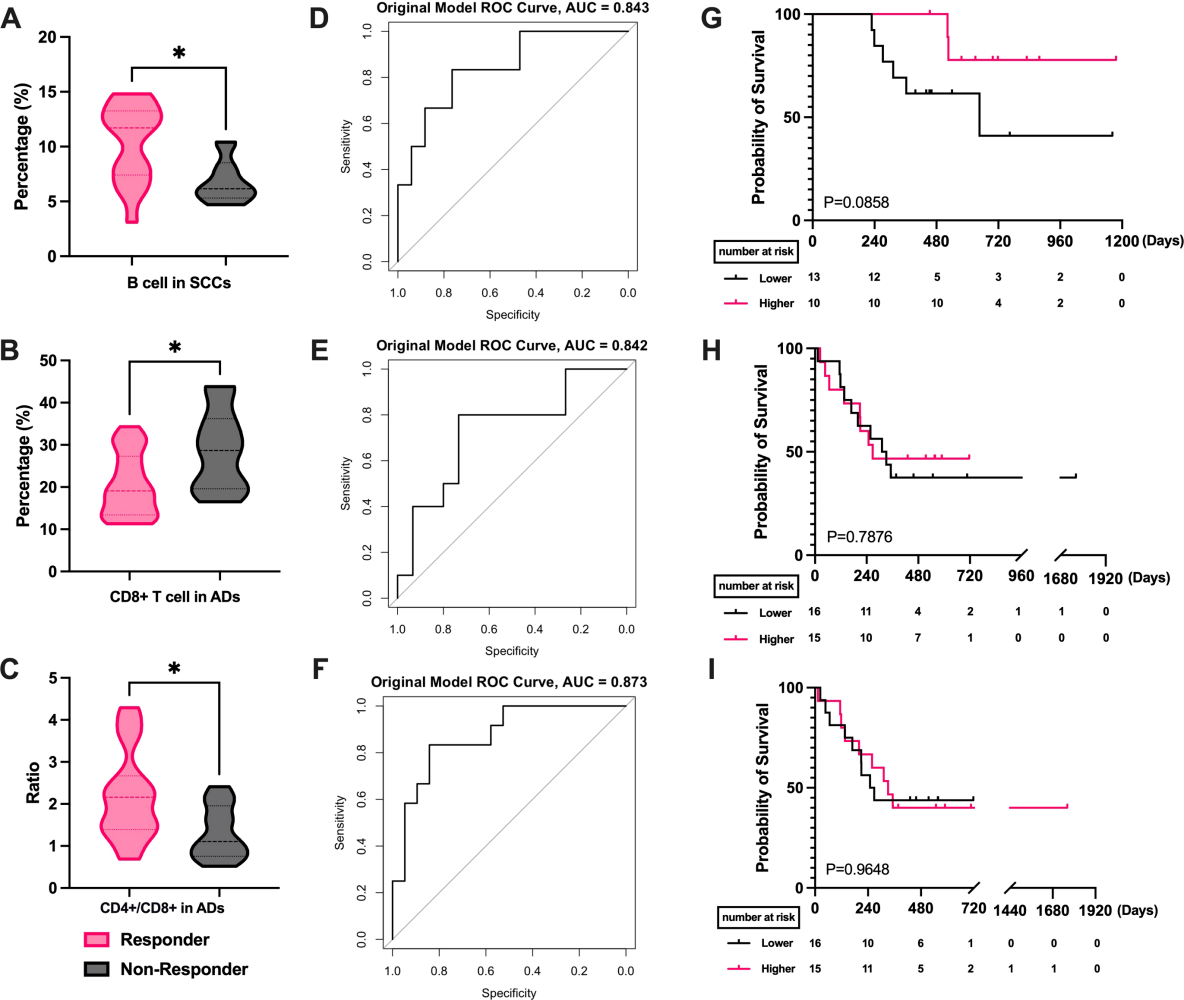
Correlation of PBLSL with the efficacy and prognosis in different pathological type groups. **(A)** B cell levels between responders and non-responders in SCCs. **(B)** CD8+ T cell levels between responders and non-responders in ADs. **(C)** CD4+/CD8+ T cell ratios between responders and non-responders in ADs. **(D)** The ROC curves of B-cell levels in SCCs. **(E)** The ROC curves of CD8+ T cell baseline levels in ADs. **(F)** The ROC curves of CD4+/CD8+ T cell ratios in ADs. **(G)** PFS between the high and low B cell level groups in SCCs. **(H)** PFS in the groups of high and low CD8+ T cell in ADs. **(I)** PFS between the high and low CD4+/CD8+ T cell ratios level groups in ADs. *p<0.05; AD, adenocarcinomas; SCC, squamous cell carcinomas; PFS, progression-free survival.

Our study included 23 cases of SCLC and 54 cases of advanced NSCLC, with 31 adenocarcinomas (AD) and 23 squamous cell carcinomas (SCC) among the NSCLC cases. We separately analyzed the correlation between pathology type and peripheral blood lymphocytes. The analysis revealed that SCC patients with higher B cell proportions had better efficacy (P = 0.0291, **Figure 4A**), while other lymphocyte subgroups were not linked to efficacy. ROC analysis showed that B cell proportion reliably predicted radiotherapy efficacy in SCC (AUC=0.843, **Figure 4D**). In contrast, in the AD group, a lower CD8+ T cells (P = 0.0119, **Figure 4B**) and higher CD4+/CD8+ T cell ratios (P = 0.0155, **Figure 4C**) correlated with better efficacy. ROC curves indicating predictive significance for CD8+ T cell levels (AUC=0.842, **Figure 4E**) and CD4+/CD8+ T cell ratios (AUC=0.873, **Figure 4F**). The mean AUC of all three cells exceeded 0.7(**Supplementary Figure S4**). However, further survival analysis found no lymphocyte subset as a reliable prognostic marker for SCC and AD (**Figures 4G–I**). In SCLC, none of the lymphocyte subsets demonstrated efficacy or prognostic predictive ability (**Supplementary Figure S5**).

### 3.4 The correlation between PBLSL and efficacy of different age groups

**Figure 5:**
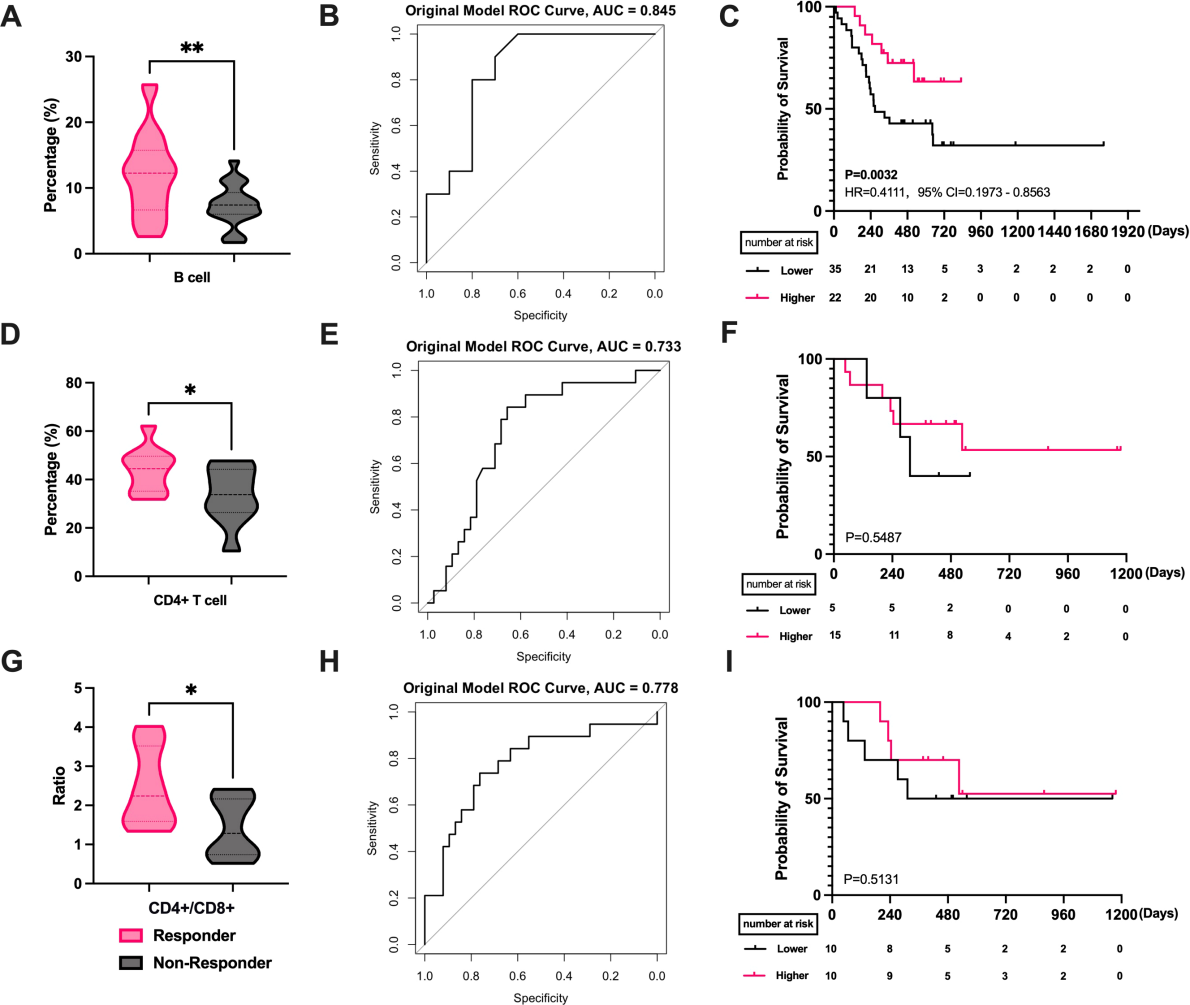
Correlation of PBLSL with the efficacy and prognosis in different age groups. **(A)** B cell level between responders and non-responders in patients under 65. **(B)** The ROC curves of B-cell baseline levels in patients under 65. **(C)** PFS between the high and low B cell level groups in patients under 65. **(D)** CD4+ T cell levels between responders and non-responders in patients over 65. **(E)** The ROC curves of CD4+T cell levels in patients over 65. **(F)** PFS between the high and low CD4+ T cell level groups in patients over 65. **(G)** CD4+/CD8+ T cell ratios between responders and non-responders in patients over 65. **(H)** The ROC curves of CD4+/CD8+ T cell ratios in patients over 65. **(I)** PFS between the high and low CD4+/CD8+ T cell ratios level groups in patients over 65. *p<0.05; **p<0.01; PFS, progression-free survival.

We studied lymphocyte subset variations by age. Among patients aged 65 or younger, higher B cell levels correlated with better efficacy (P = 0.0036, **Figure 5A**). ROC analysis indicated B cells’ potential as a treatment efficacy biomarker in this group (AUC = 0.845, **Figure 5B**). The B cell level cutoff of 11.45 was determined, dividing patients into high and low-level groups. Survival analysis showed a median PFS of 268 days in the low-level group, while not reached in the high-level group (P = 0.0332, HR = 0.4111, 95% CI = 0.1973 to 0.8563, **Figure 5C**). In patients over 65, patients with better efficacy had higher CD4+ T cell levels (P = 0.0433, **Figure 5D**) and a higher CD4+/CD8+ T cell ratios (P = 0.0338, **Figure 5G**). ROC curves for CD4+ T cell levels (AUC = 0.733, **Figure 5E**) and the CD4+/CD8+ T cell ratios (AUC = 0.778, **Figure 5H**) showed predictive significance for efficacy. Similarly, the mean AUC for all three cells was greater than 0.6(Supplementary **Figure S6**). Survival curve analysis showed no significant difference between high and low-level CD4+ T groups (P = 0.5487, **Figure 5F**) or CD4+/CD8+ T cell ratios groups (P = 0.5131, **Figure 5I**).

## 4 Discussion

In 2022, the International Agency for Research on Cancer, operating under the World Health Organization, published the most recent global cancer estimates. The findings showed that lung cancer retained its position as the most common and deadliest cancer globally. The phase III PACIFIC trial, compared durvalumab with placebo in patients with unresectable, stage III NSCLC and no disease progression after concurrent chemoradiotherapy, has reported 5-year overall survival outcomes, demonstrating a 43% overall survival rate in the durvalumab arm (25). This reality emphasizes the critical need to improve objective clinical response rates to standard treatments and the precision in selecting favorable patient populations.

Now, research on predictive biomarkers mainly focuses on imaging features, genetic detection, and liquid biopsy (26–30). Imaging technology is improving but needs to provide a complete view of immune changes. Genetic testing is invasive and may not help late-stage patients. Liquid biopsy, like circulating tumor DNA and molecular residual disease, is gaining popularity for recurrence detection and therapy response prediction. Negative molecular residual disease after chemotherapy suggests a potential cure (31). However, liquid biopsy needs improvements in sensitivity and struggles to predict outcomes for specific patients; its use is also limited by cost.

Much of the research mainly focused on the changes in peripheral blood lymphocyte levels in immunotherapies (21–24). Nonetheless, the correlation between initial peripheral blood lymphocyte levels and radiotherapy remains inadequately understood. This study aims to address this gap. Our specific analysis across different pathological types, treatments, and age Groups in advanced lung cancer patients undergoing CRT or CRIT revealed varied lymphocyte correlations. These findings collectively emphasize the significance of the research on peripheral blood lymphocyte subsets.

Classic lymphocyte subsets, including CD3+, CD4+, CD8+, B cells, and natural killer cells, play crucial roles in regulating immunity and specifically targeting tumor cells for destruction. As proficient antigen-presenting cells, B cells can initiate and amplify antigen-specific T cells, triggering anti-tumor responses. Animal models of B-cell depletion have shown a notable decline in the generation of effector-memory and IFNγ or TNFα- secreting CD4+ and CD8+ T cells, along with diminished activity of both CD4+ and CD8+ T cells in tumors and lymph nodes (32). B cells also promote the development of CD4 T follicular helper cells, enhancing the toxic effects of CD8+ T cells by producing IL-21 (33). On the other hand, peripheral B lymphocytes efficiently encoded antigen mRNA and expanded tumor antigen specific CD8 T cells *in vitro*. Additionally, B lymphocytes also mediated the presentation of plasmid antigen-induced robust Th1-biased immunity, effectively eliciting an anti-tumor response *in vivo* (34). Immune activation has been shown to depend on the collaborative relationship between B and T cells. An increase in infiltrating B cells was observed after tumors were treated with radiotherapy (35). Host plasma cells and class-switched memory B cells were highly resistant to radiotherapy (36); this suggests that the remaining B cells, after radiation, are likely to carry more potent B-cell antigen receptors responsible for the development and differentiation ensuring effective subsequent immune interactions. Our findings indicated that elevated B-cell levels correlated with better radiotherapy efficacy. Higher B-cell levels may possess a more active modulation of tumor immune changes in response to radiotherapy and immunotherapy.

CD8+ T cells are typically considered to be directly involved in anti-tumor immunity due to their ability to efficiently destroy tumors through potent cytotoxicity (37). Nevertheless, the unique role of CD4+ T cells in optimizing anti-tumor immunity is becoming increasingly well-established. CD4+ T cells are indispensable support for T-cell initiation. They facilitate the initiation of the CD8+ gene expression program through various mechanisms and enhance the clonal expansion, differentiation, and memory formation of cytotoxic T-lymphocytes (CTL) (38, 39). Second, CD4+ effector T cells can indirectly kill major histocompatibility complex (MHC)-deficient tumor cells that evade CD8+ T cell targeting, thereby independently eradicating established tumors (40). To boost CTL production, delivery, and killing activity, systemic involvement of CD4+ T cells may be necessary. The study by Li et al. demonstrated that CD4+ T cells were significantly linked to response across all lung cancer patients received radiotherapy. They also revealed that the higher CD4+/CD8+ T cell ratios before treatment was correlated with more prolonged PFS (22). Liu et al. reported that recurrence of NSCLC was associated with decreased levels of CD4+ T cells and the CD4/CD8 ratio (41). Additionally, Miao et al. identified CD4+ CD45RA-T cells in the CD4+ T cell subsets as protective and risk factors influencing the prognosis of NSCLC patients undergoing ICI (24). Patients exhibiting a notably high pre-treatment proportion of CD62Llow CD4+ T cells experienced improved treatment response and long-term remission (42). Combining radiotherapy with ICIs enhanced non-redundant immune mechanisms in anti-tumor responses, a trend observed in patients undergoing CRT and CRIT in our study. This highlights the importance of maintaining stable CD4+ T cell and the CD4+/CD8+ T cell ratios for optimal immune function. Regular monitoring of CD4+ T cell levels and the CD4+/CD8+ T cell ratios in peripheral blood could become a critical strategy for guiding treatment decisions in lung cancer patients, whether they receive radiotherapy or immunotherapy.

Conversely, our study did not find the correlation between CD8+ T cells in peripheral blood and treatment efficacy. Similarly, other studies did not observe a significant role for CD8+ T cells (22–24). Instead, the proliferation of CD8+ T cells in peripheral blood during treatment correlated with sustained clinical benefit (18). In the Impassion130 study, CD8+ TILs were identified as a stratified feature associated with improved clinical outcomes in immunotherapy (43). A meta-analysis further illustrated that increased CD8+ TIL density was a favorable prognostic indicator for ICIs therapy and combined therapy, rather than peripheral blood (44). This suggests that CD8+ T cell activity may be more significant than peripheral blood levels measured at a single time point. CD8+ T cells expand and differentiate into CTLs, migrating from peripheral blood to the tumor site to exert their effect. Elevated levels of CD8+ T cells in peripheral blood may imply a lack of lymphocyte infiltration within the tumor, indicating a weakened antagonistic effect against the tumor. We did not observe a predictive benefit of baseline NK cell levels in peripheral blood for CRT and CRIT outcomes. There is growing evidence that NK cells can display adaptive immune characteristics (45). NK cells are divided into two subsets based on CD56 expression: CD56dim and CD56bright. In peripheral blood, CD56dim NK cells comprise 90% of the population. While CD56dim NK cells have antitumor cytotoxicity, some studies have found that the cytotoxic CD56dim CD16+ NK cell subset frequency is lower in cancer patients compared to healthy individuals (46, 47). In addition, regulatory CD56bright NK cells isolated from cancer patients exhibited significantly lower NKp46 expression and showed varying degrees of positive or negative correlation with MCP-1, IP-10, VEGF, and Eotaxin (47). These results indicate that cancer patients exhibit a shift in NK characteristics with lower cytotoxicity and activation, which may be the underlying reason why NK cells have not been excellent predictors in some peripheral blood studies (23, 24, 48), including this study. A clearer understanding of the detailed phenotype and temporal changes of inhibitory lymphocyte markers could provide further insight. Considering the potential effects of cell dynamics, understanding of the detailed phenotype and continuous monitoring of lymphocyte changes during treatment could provide valuable insights.

The peak demographic for advanced lung cancer comprises the elderly, yet the survival benefits of radiotherapy remain contentious (49). Older patients (>70 years old) undergoing chemoradiotherapy were considered to exhibit poorer survival and heightened mortality during treatment (50). In contrast, recent retrospective studies suggest no disparity in survival between older and younger patients receiving chemoradiotherapy (51, 52). Age impacts the immune system, a gradual process known as immunosenescence. A peripheral blood study of 1,068 healthy individuals, including young (19-44 years), middle-aged (45-64 years), and elderly (65-80 years) participants, showed that lymphocytes decline with age, both in number and function (53). Patients with tumors experience immune compromise. A comparison of lymphocyte subpopulations in NSCLC patients and healthy individuals revealed a significant decrease in lymphocyte counts across different stages of NSCLC compared to healthy controls (54). Advanced age is accompanied by decreased T and B lymphocytes and reduced telomerase activity, ultimately weakening immune effector function (55, 56). Yang et al. observed opposing apoptotic gene expression for CD4+ and CD8+ T cells with age (57). T lymphocyte distribution may exacerbate with aging, potentially disrupting immune equilibrium. Moreover, a study found that heightened CD8+ T cell subsets expression has been linked to severe immune-related adverse reactions (24). CD8+ cells, in particular, fluctuate more strongly under age changes than CD4+ (58). Our study identified that older patients with higher CD4+ T cell levels and higher CD4+/CD8+ T cell ratios exhibited improved efficacy, while in younger patients, B-cell levels predicted efficacy. This highlights the importance of lymphocyte subset levels for treatment evaluation and their potential as key indicators for treatment screening.

Lymphocytes have been found to differentially contribute to the shaping of the tumor microenvironment and to the promotion of immune interactions between SCC and AD. Single-cell RNA sequencing revealed that SCC tissues exhibited higher infiltration of mast cells, B cells, CD8 cells, and Treg cells. In contrast, AD showed a prevalence of effector and activated cells and a lower number of Tregs (59). Our findings indicate that efficacy in SCCs correlates more strongly with B-cell levels, while in ADs, the CD4+/CD8+ T cell ratios play a more critical role in predicting efficacy; however, neither is assessed as a prognostic marker. The heterogeneity of the microenvironment may influence the variations in intratumoral recruitment of lymphocytes to the peripheral blood. Additionally, we observed no relevance of lymphocyte subsets in small-cell lung cancer. Generally, SCLC is considered to be an immune desert tumor with low MHC expression (60). Our negative results possibly suggest that immune cell infiltration is hindered in SCLC, indicating the sluggish mobilization of intratumoral or circulating lymphocyte subpopulations to exert immune effects in this cancer type.

However, there is still room for further research. First, increasing the number of patients, possibly needing more research centers, is essential to validate these findings in a larger sample population. Additionally, our continued focus on detailed immunophenotyping of cell subsets and monitoring their dynamic changes over time is necessary to better understand the potential of lymphocyte subsets as reliable biomarkers.

## 5 Conclusion

The relationship between CRT or CRIT treatment and peripheral blood immune cells is becoming better understood. We investigated the efficacy and prognostic value of CRT and CRIT in advanced lung cancer patients by analyzing peripheral lymphocyte subsets. We found varying lymphocyte subsets reflecting efficacy in distinct patient subgroups, an aspect that has received limited attention previously. Monitoring lymphocyte subsets in peripheral blood may aid in determining optimal treatment strategies for advanced lung cancer, offering tailored and personalized approaches for patients.

## Data Availability

The raw data supporting the conclusions of this article will be made available by the authors, without undue reservation.
