# Supplementary material for: Heterogeneity in peripheral blood immune lymphocyte subsets predicts the response of immunotherapy or chemoradiotherapy in advanced lung cancer: an analysis across different pathological types, treatment modalities and age
Source: Front Immunol. 2024 Oct 17;15:1464728. doi: 10.3389/fimmu.2024.1464728 (PMC11524866; doi:10.3389/fimmu.2024.1464728)
Supplement: Supplementary file 1 [file DataSheet1.docx]

**Supplementary Material**

**
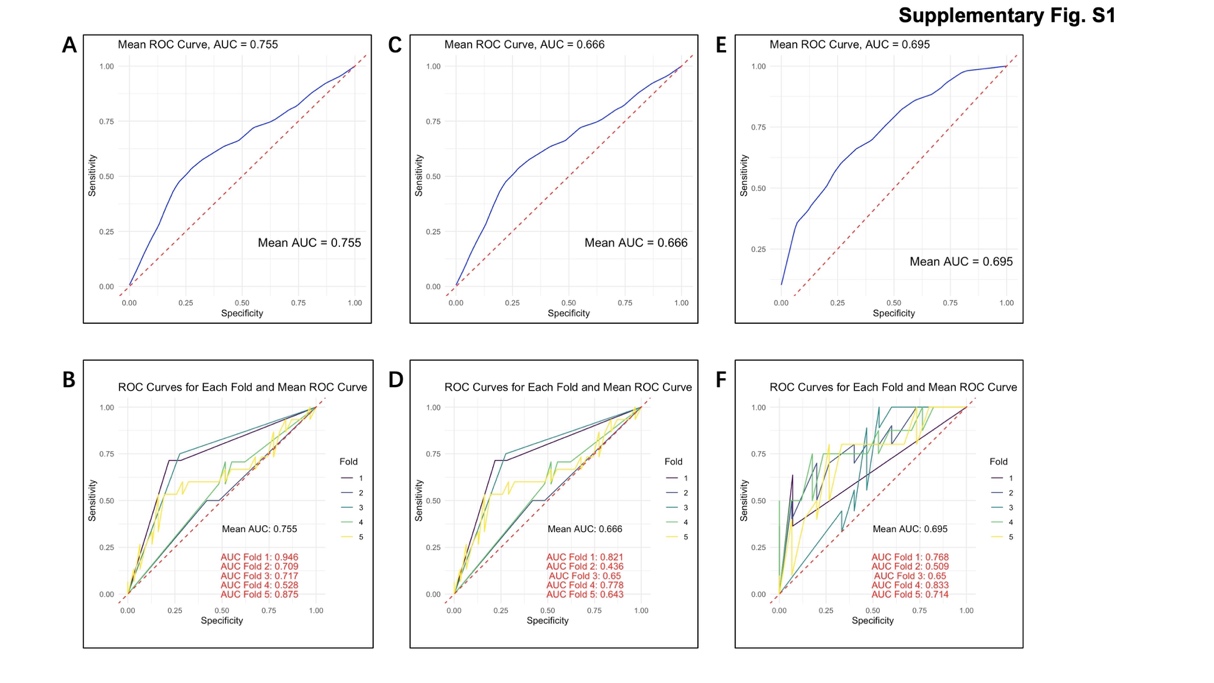
**

**Supplementary Figure 1.** Mean AUC and 5-fold cross validation of B cells, CD4+/CD8+ T cell ratio, and CD8+ T cells in all patients.

**A:** Mean AUC of B cells. **B:** 5-fold cross validation of B cells. **C:** Mean AUC of CD4+/CD8+ T cell ratio. **D:** 5-fold cross validation of CD4+/CD8+ T cell ratio. **E:** Mean CD8+ T cell AUC. **F:** 5-fold cross validation of CD8+ T cells.

**
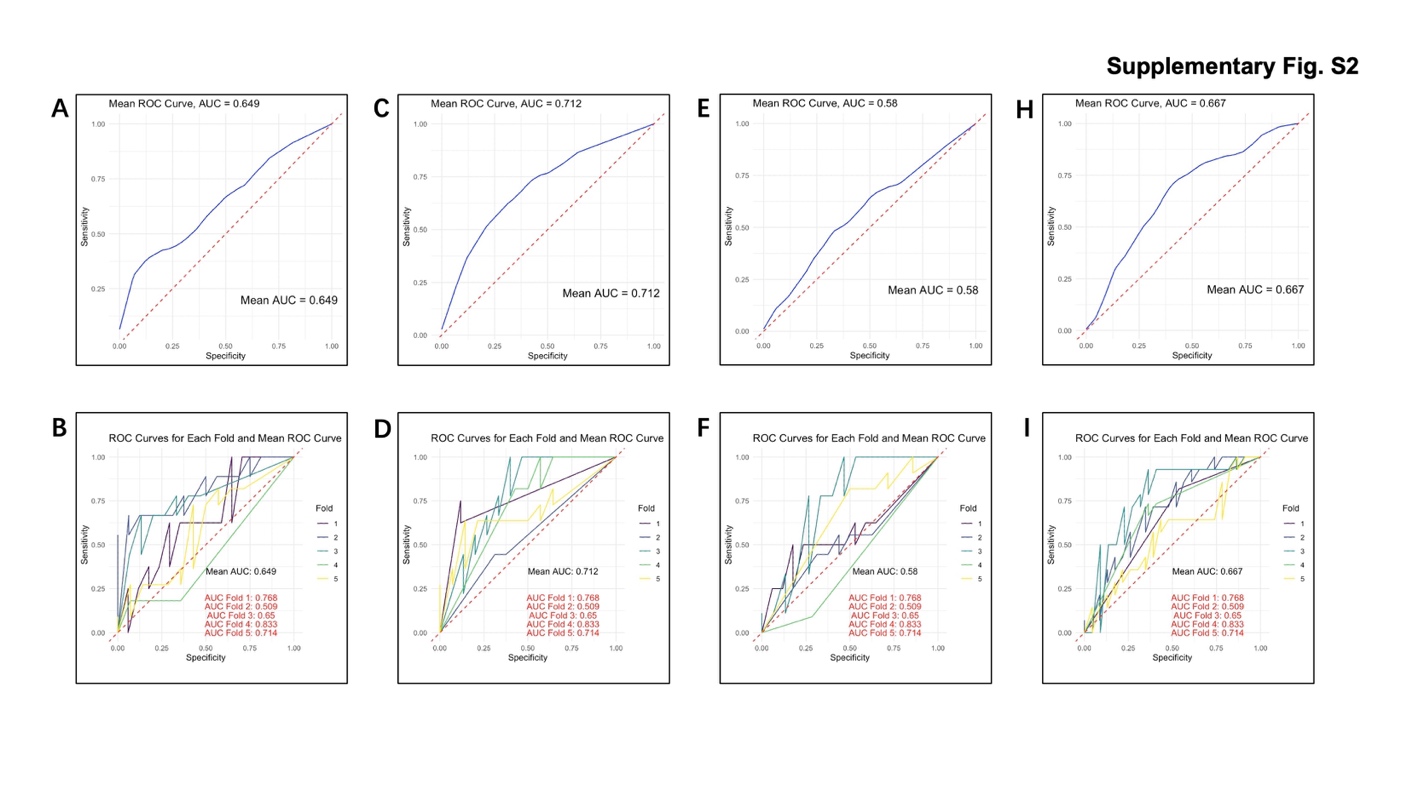
 Supplementary Figure 2.** Mean AUC and 5-fold cross-validation of PBLSL in different treatment modalities.

**A:** Mean AUC of CD4+ T cells in CRIT. **B:** 5-fold cross-validation of CD4+ T cells in CRIT.**C:** Mean AUC of CD4+/CD8+ T cell ratio in CRIT. **D:** 5-fold cross-validation of CD4+/CD8+ T cell ratio in CRIT. **E:** Mean AUC of CD8+ T cells in CRIT. **F:** 5-fold cross-validation of CD8+ T cells in CRIT. **H:** In CRT, mean AUC of B cells. **I:** 5-fold cross validation of B cells in CRT.

**PBLSL, peripheral blood lymphocyte subsets level; CRT, chemoradiotherapy; CRIT, chemoradiotherapy and immunotherapy**

**
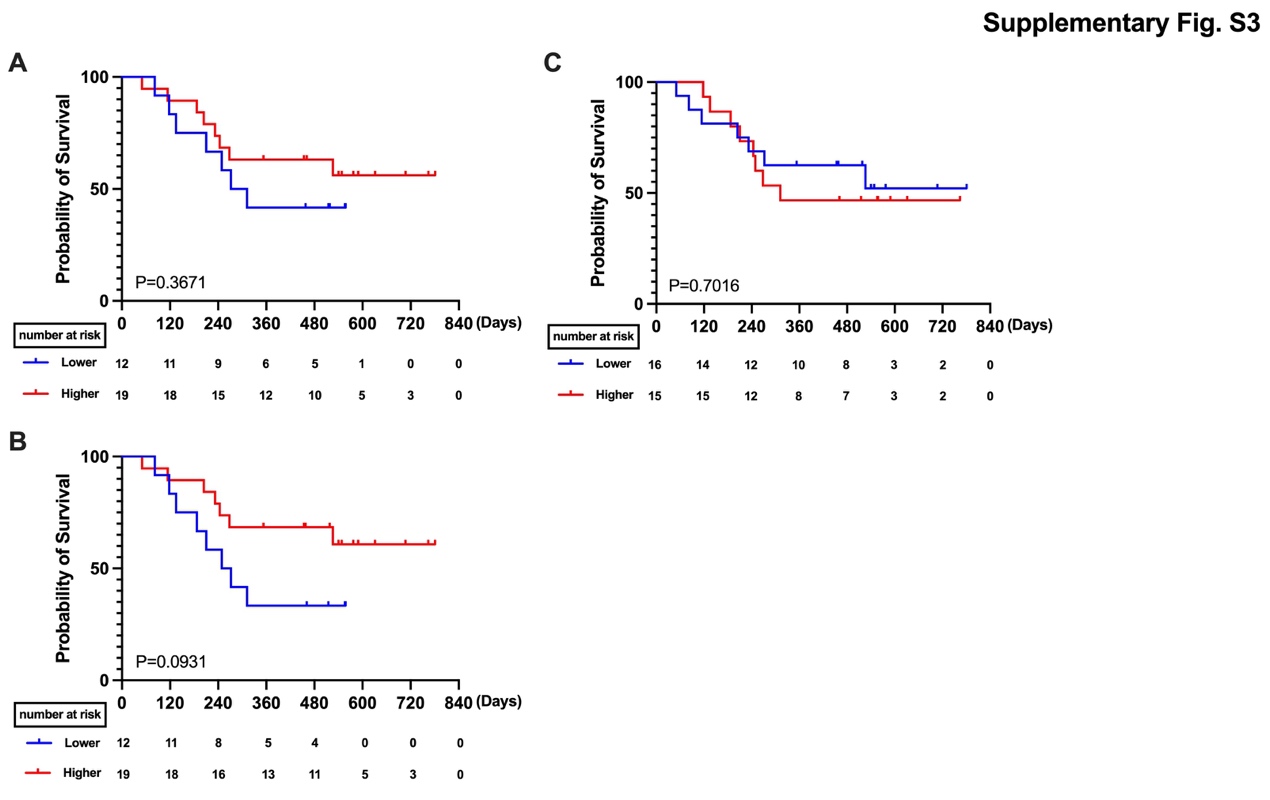
** **Supplementary Figure 3.** Prognostic analysis of PBLSL differences in patients undergoing IRT

**A:** PFS in the groups of high and low CD4+ T cell. **B:** PFS in the groups of high and low CD8+ T cell. **C:** PFS in the groups of high and low CD4+/CD8+ T cell ratios.

**PFS, progression-free survival**

**
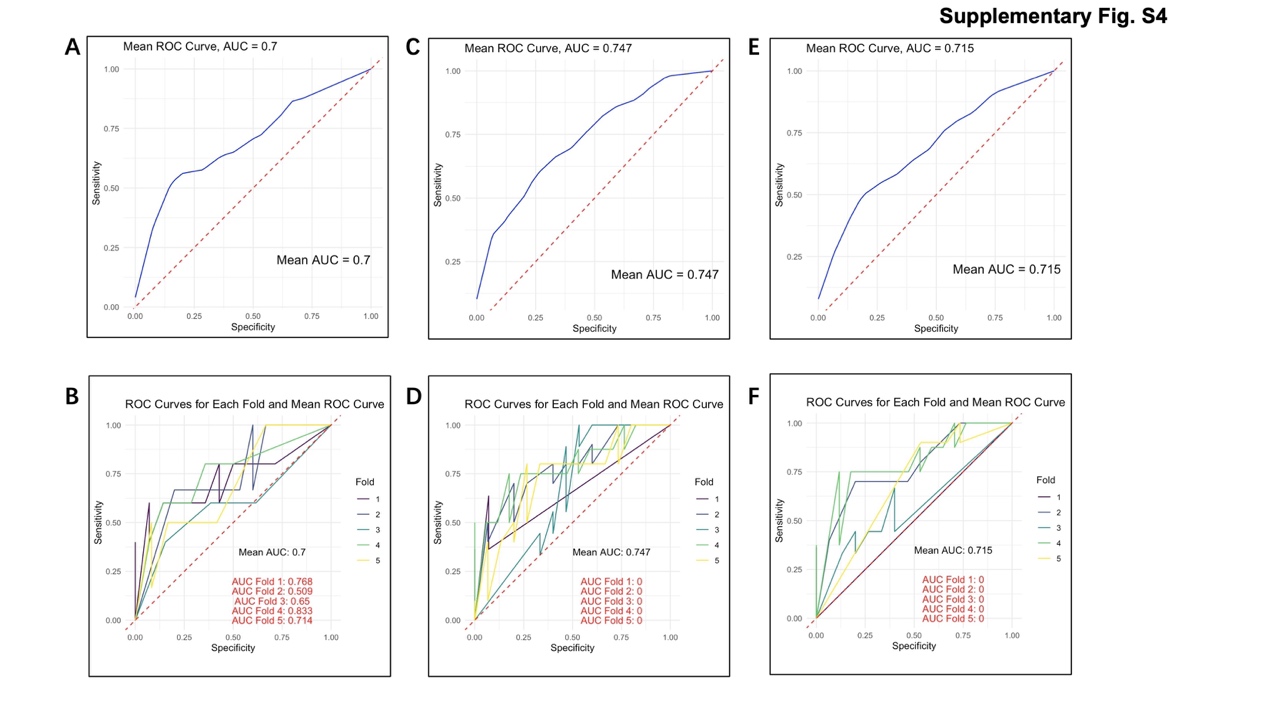
**

**Supplementary Figure 4.** Mean AUC and 5-fold cross-validation of PBLSL in different pathologic types.

**A:** Mean AUC of B cells in SCCs. **B:** 5-fold cross-validation of B cells in SCCs. **C:** Mean AUC of CD8+ T cells in AD. **D:** 5-fold cross-validation of CD8+ T cell ratio in AD. **E:** Mean AUC of CD4+/CD8+ T cell ratio in AD. **F:** 5-fold cross-validation of CD4+/CD8+ T cell ratio in AD.

**SCC, Squamous cell carcinoma; AD, Adenocarcinoma**


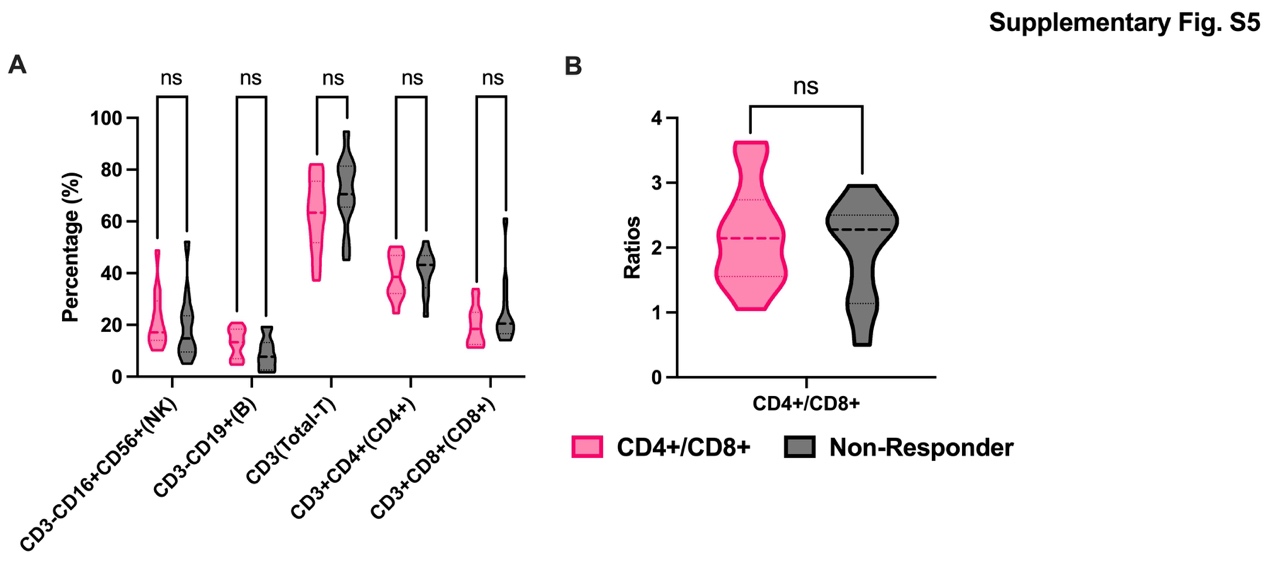


**Supplementary Figure 5.** PBLSL between responders and non-responders in SCLC.

**A:** PBLSL between responders and non-responders of SCLCs. **B:** The ratios of CD4+/CD8+ in SCLCs.

**ns, no significance; SCLC, small cell lung cancer.**

**
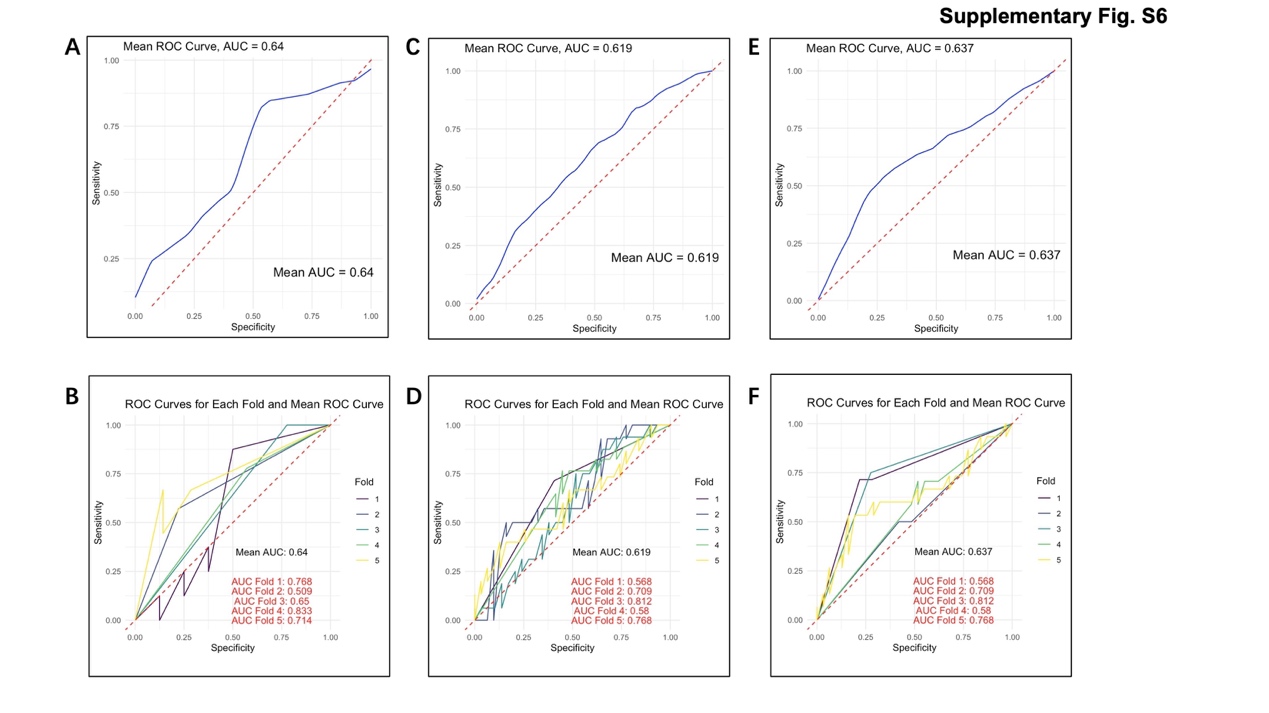
 Supplementary Figure 6.** Mean AUC and 5-fold cross-validation of PBLSL in different ages.

**A:** Mean AUC of B cells in patients under 65. **B:** 5-fold cross-validation of B cells in patients under 65. **C:** Mean AUC of CD4+ T cells in patients over 65. **D:** 5-fold cross-validation of CD4+ T cell ratio in patients over 65. **E:** Mean AUC of CD4+/CD8+ T cell ratio in patients over 65. **F:** 5-fold cross-validation of CD4+/CD8+ T cell ratio in patients over 65.
